# The ecdysis triggering hormone system is essential for successful moulting of a major hemimetabolous pest insect, *Schistocerca gregaria*

**DOI:** 10.1038/srep46502

**Published:** 2017-04-18

**Authors:** Cynthia Lenaerts, Dorien Cools, Rik Verdonck, Lina Verbakel, Jozef Vanden Broeck, Elisabeth Marchal

**Affiliations:** 1 Molecular Developmental Physiology and Signal Transduction, KU Leuven, Naamsestraat 59, P.O. Box 02465, B-3000 Leuven, Belgium

## Abstract

Insects are enclosed in a rigid exoskeleton, providing protection from desiccation and mechanical injury. To allow growth, this armour needs to be replaced regularly in a process called moulting. Moulting entails the production of a new exoskeleton and shedding of the old one and is induced by a pulse in ecdysteroids, which activates a peptide-mediated signalling cascade. In Holometabola, ecdysis triggering hormone (ETH) is the key factor in this cascade. Very little functional information is available in Hemimetabola, which display a different kind of development characterized by gradual changes. This paper reports on the identification of the ETH precursor and the pharmacological and functional characterisation of the ETH receptor in a hemimetabolous pest species, the desert locust, *Schistocerca gregaria*. Activation of *Schgr*ETHR by *Schgr*ETH results in an increase of both Ca^2+^ and cyclic AMP, suggesting that *Schgr*ETHR displays dual coupling properties in an *in vitro* cell-based assay. Using qRT-PCR, an in-depth profiling study of *SchgrETH* and *SchgrETHR* transcripts was performed. Silencing of *SchgrETH* and *SchgrETHR* resulted in lethality at the expected time of ecdysis, thereby showing their crucial role in moulting.

An important reason why insects have been so undeniably successful on this planet is the protection provided to them by the presence of a rigid exoskeleton or cuticle. This cuticle protects them from desiccation and mechanical injury, but also determines their shape and coloration, and it provides a base to which muscles are attached. Although the rigidity of this cuticle protects them, it renders the apparently ‘simple’ process of growing extremely complex. For allowing the insect to increase in volume, it must be shed periodically and a larger one must be synthesized. This process is called moulting and involves the synthesis of a new cuticle underneath the old one, the digestion and resorption of most of the old cuticle, and finally the shedding of the old cuticle in a behavioural process designated as “ecdysis”^1^. Ecdysis is not only the gateway to the next developmental stage, it represents a very vulnerable phase repeated several times during insect development. Failure to successfully complete ecdysis will in most cases result in death, making this process an excellent target in the search for new insect pest management strategies.

In insects, successful moulting depends on two lipophilic hormone families: juvenile hormones (JHs) and ecdysteroids. While JHs determine the nature of the moult, ecdysteroids trigger the moulting process and activate a neuropeptide signalling cascade which in turn regulates the ecdysis sequence at the end of the moulting cycle. Models on the combined actions of hormonal and neural factors regulating insect ecdysis behaviour have been conceptualized based on research performed in the Holometabola *Manduca sexta, Drosophila melanogaster, Bombyx mori* and *Tribolium castaneum*^2^,^3^,^4^,^5^,^6^,^7^,^8^,^9^,^10^,^11^,^12^,^13^,^14^,^15^,^16^,^17^. For a detailed and comprehensive review on the neuroendocrine regulation of ecdysis the reader is referred to Žitňan and Adams^18^. In short, in *M. sexta*, increasing concentrations of ecdysteroids result in production of ecdysis triggering hormone (ETH) in Inka cells attached to the trachea. High ecdysteroid concentrations will however prevent the release of ETH from these cells. On the other hand, high ecdysteroid levels sensitize the central nervous system (CNS) to ETH by expressing the ETH receptor (ETHR). When ecdysteroid titres decline again, ETH is secreted and activates neurons producing kinins and Corticotropin Releasing Factor (CRF)-like diuretic hormones (DHs), which trigger the pre-ecdysis behaviour sequence. At the end of pre-ecdysis ETH acts on the ventromedial eclosion hormone (EH) neurons, resulting in the release of EH. Via a positive feedback loop between EH and ETH there is a massive release of both peptides in the hemolymph, which leads to the activation of the neuronal network producing cyclic GMP (cGMP), crustacean cardioactive peptide (CCAP), bursicon and other neuropeptides which control ecdysis and post-ecdysis behavioural sequences. Studies in Holometabola have provided a consensus model in the regulation of ecdysis and at the same time have underlined clear differences between species. For instance, in *M. sexta* and *T. castaneum* CCAP is a crucial trigger for the ecdysis behavioural sequence, while it does not seem to play a role in moulting in *D. melanogaster*^19^. Obtaining information on the regulation of ecdysis behaviour in a wider range of insect taxa can help improve our current understanding of the evolutionary processes that resulted in the existing array of innate ecdysis behaviours and their endocrine regulation.

ETH, the key triggering factor in the neuropeptidergic cascade regulating ecdysis^15^,^20^, was first discovered in *M. sexta*^16^. Cloning of the *M. sexta eth* gene revealed that it not only codes for the ETH peptide, but also for the pre-ecdysis triggering hormone (PETH). In other insects the *eth* gene usually also encodes two peptides, ETH1 and ETH2^4^,^14^,^21^,^22^. ETH receptors were first discovered in *D. melanogaster*^8^ and have also been pharmacologically and functionally characterized in *M. sexta, T. castaneum* and *Aedes aegypti*^4^,^10^,^21^,^23^. The *ethr* gene in these species encodes two ETHR subtypes that are generated by alternative splicing of the 3′ exon (ETHR-A and ETHR-B). ETHR-A and ETHR-B are G protein-coupled receptors (GPCRs) and, depending on the species, these can have a different affinity for their endogenous ligands, ETH1 and ETH 2 or in case of *M. sexta* PETH and ETH. Silencing of *ETH* or *ETHR* in different Holometabola resulted in lethality at the expected time of ecdysis^4^,^5^,^8^,^15^,^20^.

Our present study focusses on the pharmacological and functional characterization of the ETH peptide system in a hemimetabolous insect, the desert locust, *Schistocerca gregaria*. This ravenous swarm-forming phytophagous pest insect forms a real threat to the agricultural production in some of the world’s poorest countries. To our knowledge, we are the first to investigate this key factor in the development of a hemimetabolous insect species. We were able to clone the cDNAs for the ETH precursor (*Schgr*ETHpre) encoding the two ETHs and receptor (*Schgr*ETHR). After showing that *Schgr*ETH1 and *Schgr*ETH2 can activate *Schgr*ETHR, we further investigated the downstream signalling pathway by expressing the receptor in different cell lines. Furthermore, we report on an in-depth transcript profiling study of *SchgrETHR* and *SchgrETHpre*. Moreover, RNA interference (RNAi)-mediated silencing of *SchgrETH* or its receptor resulted in lethality at the expected time of ecdysis, demonstrating their crucial role in the development of *S. gregaria*. Given that ETHR is a GPCR, and many compounds are known to target surface receptors, we believe that ETHR is an excellent candidate target for the development of a next generation of pest control agents.

## Results

### Cloning and sequence analysis of *SchgrETHR* and *SchgrETHpre*

**Figure 1:**
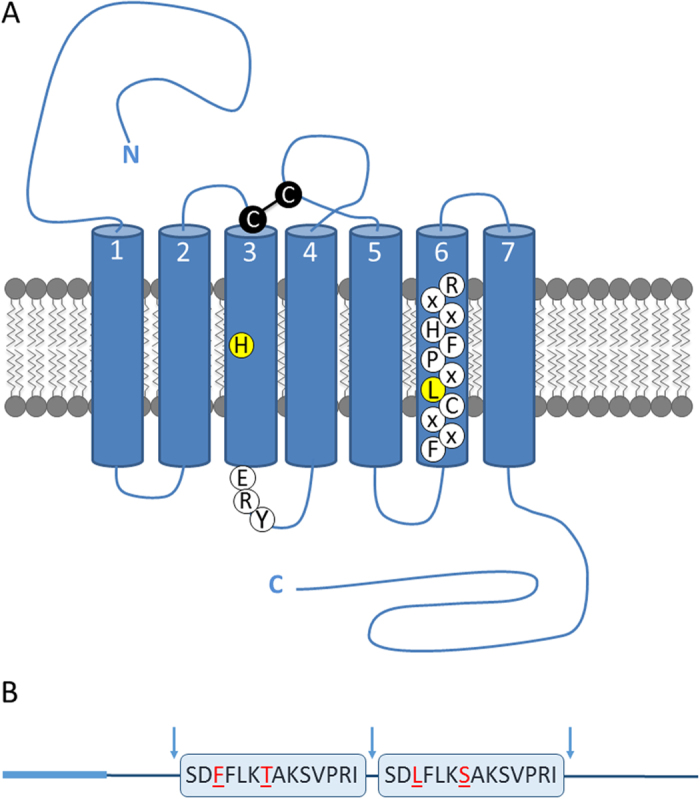
Schematic representation of *Schgr*ETHR and *Schgr*ETH precursor. (**A**) *Schgr*ETHR is a rhodopsin-like GPCR with seven transmembrane regions. The highly conserved disulphide bridge, formed between a cystein (C) at the beginning of transmembrane 3 and in extracellular loop 2, is indicated in black. Following residues are accentuated: the histidine (H) typically conserved in transmembrane region 3 of all ETH receptors, the typical rhodopsin-like conserved ERY motif immediately following the third transmembrane helix and the leucine (L) replacing tryptophan (W), which is a typical feature of ETH receptors, in the highly conserved FXXCLXPFHXXR motif in transmembrane 6. (**B**) The AA sequence of two *Schgr*ETH peptides are boxed, red residues differ between both peptides. Cleavage sites are indicated with arrows, while the signal peptide is emphasized by a thicker light blue line.

The complete open reading frames (ORF) of the *SchgrETHR* and *SchgrETHpre* found in an unpublished (in-house) *S. gregaria* transcriptome database were verified by Sanger sequencing of the PCR amplicons obtained using the primers listed in Table 1. For *Schgr*ETHR only one splice variant was found. The ORF of the *SchgrETHR* consists of 1323 nucleotides encoding a 441 amino acid-long receptor 1(Supplementary Fig. S1). Transmembrane topology prediction revealed the presence of seven hydrophobic regions forming the α-helical transmembrane segments (TM1-7) characteristic of GPCRs^24^,^25^. BLASTx searches revealed similarities of the cloned *SchgrETHR* with other insect ETHRs, vertebrate thyrotropin-releasing hormone (TRH) or ghrelin (previously known as growth hormone-secretagogue^26^) receptors. The *Schgr*ETHR sequence can be found in a multiple sequence alignment with other functionally confirmed insect ETH receptors in Supplementary Fig. S1. *Schgr*ETHR contains some of the typical features of rhodopsin-like receptors such as the ERY motif near the intracellular site of TM3 and two conserved cysteines, at the start of TM3 and in extracellular loop 2, forming a disulphide bridge ensuring proper orientation of the second extracellular loop. Also the highly conserved motif in TM6, FXXCLXPFHXXR, can be observed. In this motif, involved in GPCR activation, a leucine (L) replaces the conserved tryptophan (W) which is a typical feature of ETH receptors^27^ (Fig. 1, Supplementary Fig. S1).

The ETH precursor sequence contains a signal peptide, predicted by SignalP 4.1^28^, and several dibasic cleavage sites (Supplementary Fig. S2). The sequence contains two peptides having a C-terminal -PRIamide, which is a conserved motif for ETH peptides (-PRVamide in *Manse*PETH and *Bommo*PETH; -PRMamide in *Manse*ETH and *Bommo*ETH; -PRIamide in *Drome*ETH2, *Aedae*ETH1 and *Aedae*ETH2; -PRLamide in *Drome*ETH1)^4^,^14^,^21^,^22^. *SchgrETHR* and *SchgrETHpre* sequences have been submitted to GenBank of the National Center for Biotechnology Information (NCBI) and have received accession numbers KY129672 and KY129673, respectively.

### Pharmacological characterization of *SchgrETHR*

**Figure 2:**
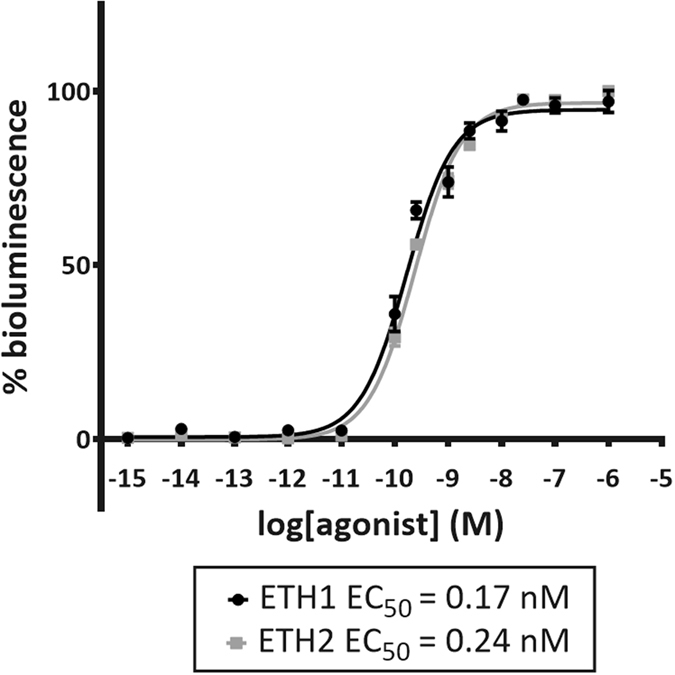
Dose-response curves for bioluminescence induced by *Schgr*ETH1 and *Schgr*ETH2 in *SchgrETHR*- expressing CHO-WTA11 cells. CHO-WTA11 cells are stably co-expressing a promiscuous Gα_16_ subunit and apo-aequorin. Each data point represents the mean (±SEM) of two independent experiments performed in triplicate. Receptor activation is shown as relative (%) to the highest value (100%) after normalization to the maximum calcium response. All values were corrected for the negative control (BSA only).

**Figure 3:**
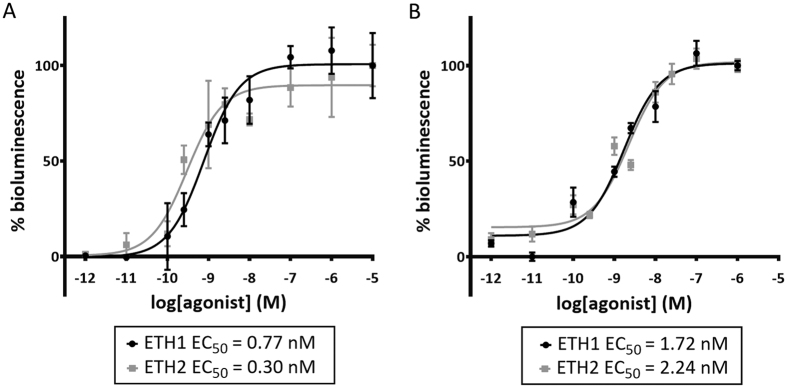
Intracellular signalling in *SchgrETHR*-expressing CHO-PAM28 and HEK293T cells. Receptor activation is shown as relative (%) to the highest value (100%) after normalization to the maximum response. Data represent the mean (±SEM) of three independent measurements, each performed in triplicate. (**A**) Dose-response curves for bioluminescence induced by *Schgr*ETH1 and *Schgr*ETH2 in *SchgrETHR*-expressing CHO-PAM28 cells. Zero response level is measured in cells challenged with BSA only. (**B**) Dose-response curves for luciferase bioluminescence induced by *Schgr*ETH1 and *Schgr*ETH2 in *SchgrETHR*-expressing HEK293T cells. Zero response level is measured in cells challenged with DMEM/IBMX.

A cell-based receptor activation assay was performed to determine whether *Schgr*ETH1 and *Schgr*ETH2 can activate the recombinant *Schgr*ETHR. *Schgr*ETHR-expressing Chinese hamster ovary (CHO-WTA11) cells stably overexpressing a promiscuous Gα_16_ subunit and apo-aequorin responded to *Schgr*ETH1 and *Schgr*ETH2 with half maximal effective concentration (EC_50_) values of 0.17 nM and 0.24 nM respectively (Fig. 2). No response was observed in cells transfected with an empty vector, indicating that *Schgr*ETH1 and *Schgr*ETH2 cannot activate endogenous receptors in the CHO-WTA11 cells. Intracellular signalling of *Schgr*ETHR was investigated in *Schgr*ETHR-expressing cells lacking the promiscuous Gα_16_ subunit (CHO-PAM28 cells) or co-expressing a cyclic AMP (cAMP) response element (CRE)-luciferase reporter (human embryonic kidney (HEK) 293 T). Both Ca^2+^ and cAMP levels increased upon challenging the ETHR-expressing cells with increasing concentrations of *Schgr*ETH1 and *Schgr*ETH2 (Fig. 3), showing that the *Schgr*ETH receptor has the capacity to signal through both second messengers.

### Transcript level studies

**Figure 4:**
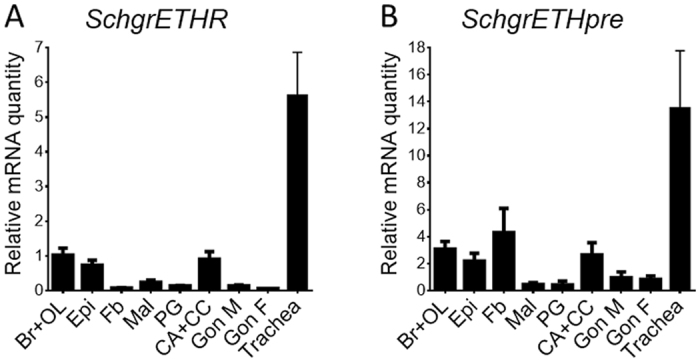
Tissue distribution profile of *SchgrETHR* and *SchgrETHpre* in fifth instar *S. gregaria.* Relative transcript levels of *SchgrETHR* and *SchgrETHpre* were measured in different fifth instar tissues using qRT-PCR. Tissues were dissected from 8-day-old male fifth instar locusts, except for the female gonads. The data represent mean ± S.E.M. of three independent pools of five animals, run in duplicate and normalized to *RP49* and *EF1α* transcript levels. Abbreviations X-axis: Br + OL: brain + optic lobes; Epi: epidermis; Fb: fat body; Mal: Malpighian tubules; PG: prothoracic glands; CA + CC: corpora allata + corpora cardiaca; Gon M: male gonads; Gon F: female gonads.

**Figure 5:**
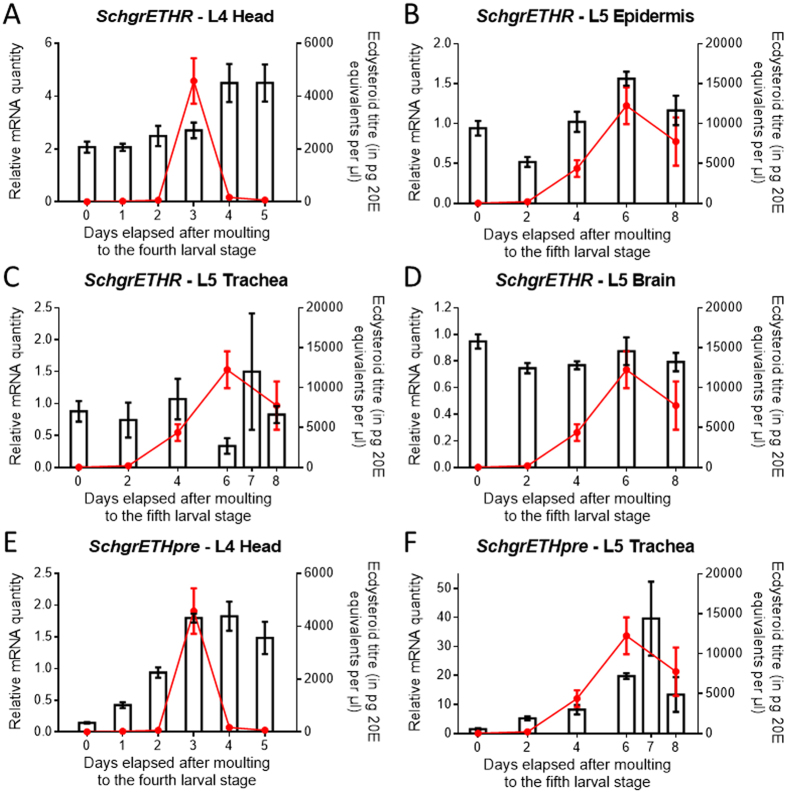
Temporal expression profile of *SchgrETHR* and *SchgrETHpre* as well as hemolymph ecdysteroid titres in fourth and fifth instar *S. gregaria*. Using qRT-PCR, the relative transcript levels of *SchgrETHR* and *SchgrETHpre* (bars) were measured every day in the heads during fourth instar development and every other day in the trachea during fifth instar development. *SchgrETHR* transcript levels were also measured every other day in the epidermis and brain during fifth instar development. The data during fourth instar development represent mean ± S.E.M. of six independent pools of three animals, run in duplicate and normalized to *RP49* and *EF1α* transcript levels. The data during fifth instar development represent mean ± S.E.M. of three independent pools of five animals, run in duplicate. The data in the epidermis were normalized to *RP49* and *EF1α,* the data in the brain were normalized to *GAPDH* and *CG13220*, and the data in the trachea were normalized to *α-tubulin1A, β-actin* and *EF1α* transcript levels. Ecdysteroid titres (red line), expressed in pg 20E equivalents per μl hemolymph, throughout the fourth and fifth instar stage were measured with an EIA. The data represent mean ± S.E.M. of five pools of three hemolymph samples taken from different animals.

Tissue-specific and temporal transcript profiles of *SchgrEHTR* and *SchgrETHpre* were determined using qRT-PCR. The tissue distribution has been studied in fifth instar locusts (Fig. 4), while the temporal distribution profile of *SchgrETHR* and *SchgrETHpre* has been studied throughout both fourth and fifth instar development (Fig. 5). Relative transcript levels of *SchgrETHR* (Fig. 5A–D) and *SchgrETHpre* (Fig. 5E,F) were measured every day in the heads during fourth instar development and every other day in the epidermis, trachea and brain during fifth instar development, both starting on the day of moulting to each stage (day 0). Under standard breeding conditions the fourth instar stage lasts for 6 days, while the fifth and last instar stage lasts for 9 to 10 days. In the same animals, hemolymph ecdysteroid levels were determined by means of EIA.

Tissue distributions of *SchgrETHR* and *SchgrETHpre* in fifth instar locusts are given in Fig. 4(A,B). The highest expression levels of both genes of interest were found in the trachea. Significantly lower expression was found in the brain and optic lobes, the epidermis and the corpora allata-corpora cardiaca (CA-CC) complex.

The temporal transcript profile of *SchgrETHR* in the heads of fourth instars remained stable until the ecdysteroid peak on day 3, after which it increased (Fig. 5A). In the epidermis of fifth instars the *SchgrETHR* transcript profile correlated with the ecdysteroid titre in the hemolymph, with a peak on day 6 (Fig. 5B). On the other hand, a relatively stable expression of *SchgrETHR* was observed in the trachea and brain of fifth instars (Fig. 5C,D). In the heads of fourth instars, the temporal distribution profile of *SchgrETHpre* increased until day 3, when a peak in ecdysteroid titres was observed, after which it remained high on day four and lowered a bit on day 5 (Fig. 5E). When looking at the temporal expression profile of *SchgrETHpre* in the trachea of fifth instars, a huge increase towards the end of this stage could be observed (Fig. 5F).

### RNA interference of *SchgrETHR* and *SchgrETHpre*

#### Knockdown efficiency

To investigate the efficiency of the RNAi-mediated knockdown of *SchgrETHR* or *SchgrETHpre,* one-day-old fourth instar locusts were injected with dsRNA (400 ng) against *SchgrETHR* or *SchgrETHpre.* Some of the locusts were sacrificed on day 4 to quantify the knockdown in the fourth instar stage. The other locusts received boost injections (400 ng) on day 4 of the fourth instar stage, as well as on day 1, 3 and 5 of the fifth instar stage. These locusts were sacrificed on day 6 of the fifth instar stage to investigate the knockdown efficiency in this developmental stage.

In both developmental stages, a significant reduction of *SchgrETHR* and *SchgrETHpre* transcript levels was observed (Suppl. Fig. S3). A reduction of 32% and 77% in relative mRNA levels of respectively *SchgrETHR* and *SchgrETHpre* could be measured in fourth instar heads (Suppl. Fig. S3A,B). In fifth instar heads, transcript levels of *SchgrETHR* and *SchgrETHpre* were respectively 65% and 71% lower than in control locusts (Suppl. Fig. S3C,D). It can be concluded that *SchgrETHR* and *SchgrETHpre* downregulation was successful in both developmental stages.

#### Effect on moulting

**Figure 6:**
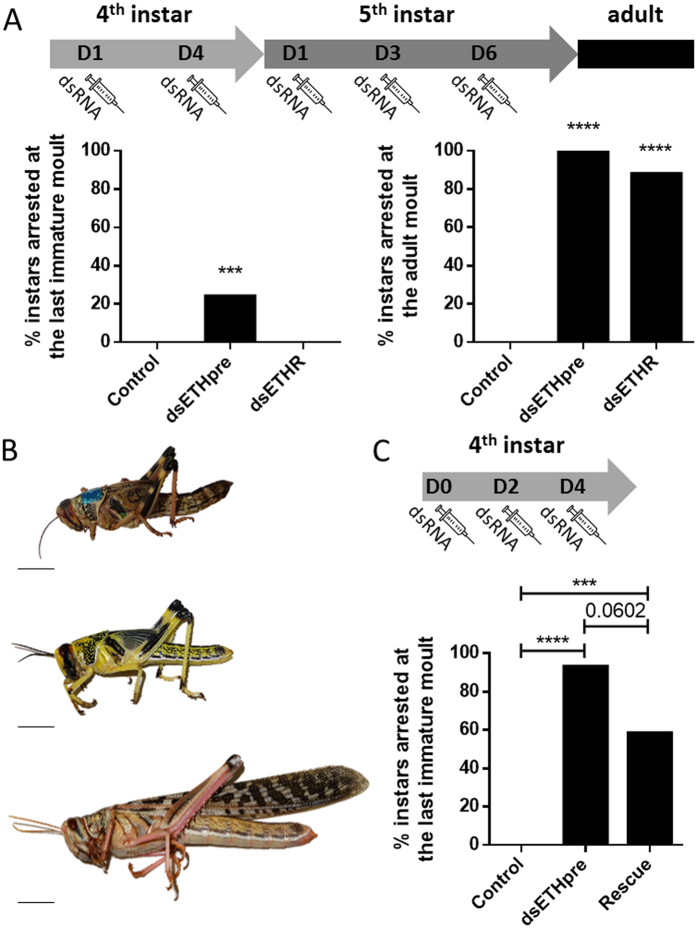
Effect of RNAi-mediated knockdown of *SchgrETHR* or *SchgrETHpre* on the last immature and adult moult of *S. gregaria*. (**A**) Percentage of instars arrested at the last immature and adult moult (N = 17–19). Locusts were injected on day 1 and 4 of the fourth instar stage, as well as on day 1, 3 and 5 of the fifth instar stage with 400 ng of dsRNA against *GFP* (control), *SchgrETHR* or *SchgrETHpre*. (**B**) Phenotypes correlated with the arrested moult at the expected timing of ecdysis. Top: RNAi phenotype (weakened and died); Middle and bottom: respectively control fifth instar and adult locusts. (**C**) Percentage of instars arrested at the last immature moult (N = 12–15). Locusts were injected on day 0, 2 and 4 of the fourth instar stage with 400 ng of dsRNA against *GFP* (control) or *SchgrETHpre* (ds*Schgr*ETHpre and rescue). To rescue the knockdown of *SchgrETHpre,* locusts were injected with 10 ng of ETH1 and ETH2 once a day from day 0 till day 3 and with 100 ng of both peptides twice a day from day 4 until the day they moulted or died. Control and ds*Schgr*ETHpre locusts were injected with *Schistocerca* Ringer according to the same injection scheme. Significant differences (p < 0.001 and p < 0.0001) are indicated by asterisks (*** and **** respectively) (Fisher’s exact test).

To investigate the role of *SchgrETHR* and *SchgrETHpre* in the moulting process, a similar injection scheme as described in the previous paragraph was applied, except for the boost injection on day 5 of the fifth instar stage, which was replaced by an injection on day 6 of this final instar stage. While 24% (4/17) of the ds*SchgrETHpre* fourth instar locusts were arrested at their expected time of ecdysis, all (17/17) of the ds*SchgrETHR* locusts and 95% (18/19) of the control locusts successfully moulted to the last instar stage. For the adult moult on the other hand, 88% (15/17) and 100% (13/13) of the respectively ds*SchgrETHR* and ds*SchgrETHpre* fifth instar locusts were not able to complete ecdysis, compared to control locusts that all (18/18) successfully moulted to the adult stage (Fig. 6A). The observed phenotype for the developmentally arrested knockdown locusts was similar for both target genes in both fourth and fifth instars (shown for fifth instars in Fig. 6B). At the expected time of ecdysis (around day 9 of the final instar), the ds*SchgrETHR* or ds*SchgrETHpre* knockdown locusts became pink, like control locusts right before moulting, but then they weakened, darkened and eventually died (Fig. 6B, top).

Several unsuccessful attempts were made to rescue the effect of the RNAi-mediated knockdown of *SchgrETHpre* in fifth instars by injecting ETH1 and ETH2. Neither varying the timing of injection of both peptides, nor changing the amount of injected peptides, resulted in rescuing the ds*SchgrETHpre* knockdown fifth instars. Since more variation exists in the timing of ecdysis from fifth instar to adult than from fourth to fifth instar, we optimized the dsRNA injection scheme in fourth instars in order to have a higher number of developmentally arrested instars at the last immature moult. When injecting dsRNA against *SchgrETHpre* on the day of moulting to the fourth instar stage (day 0), as well as on day 2 and 4, 93% (14/15) of the knockdown locusts were arrested during the last immature moult (Fig. 6C). Also in this stage we had to optimize the timing of injection and the amount of injected peptides. We first tried to inject different amounts of both peptides at the end of the fourth instar, but this did not result in a successful rescue. The best results to rescue the knockdown phenotype were obtained with following ETH injection scheme: injection of 10 ng of ETH1 and ETH2 once a day from day 0 till day 3, followed by injecting 100 ng of both peptides twice a day from day 4 until the day they moulted/died. With this injection scheme we were able to rescue 41% (5/12) of the ds*SchgrETHpre* knockdown locusts (Fig. 6C).

### Effect of silencing *SchgrEcR*/*SchgrRXR* on transcript levels of *SchgrETHR a*nd *SchgrETHpre*

**Figure 7:**
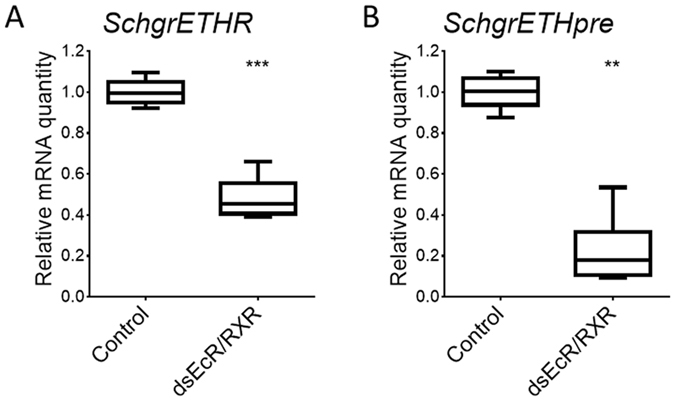
Effect of RNAi-mediated knockdown of the ecdysone receptor complex, *SchgrEcR*/*SchgrRXR*, on the transcript levels of *SchgrETHR* and *SchgrETHpre* in 6-day-old fifth instar male *S. gregaria*. Relative transcript levels were measured in the epidermis from control locusts and locusts injected with *SchgrEcR* and *SchgrRXR* dsRNA using qRT-PCR. Newly emerged fifth instar locusts were injected with 200 ng of *SchgrEcR* and *SchgrRXR* dsRNA. A boost injection was given three days later. The data represent box plots (min to max) of six independent pools of three animals, run in duplicate and normalized to *β-actin* and *EF1α* transcript levels. Significant differences (p < 0.0001) are indicated by asterisks (****) (two-sided Welch’s *t*-test on log-transformed data).

To determine whether ETH and its receptor belong to the neuropeptidergic cascade that is induced by a peak in ecdysteroid titre, newly moulted fifth instar locusts were injected with dsRNA (200 ng) against both components of the ecdysone heterodimeric receptor complex, *SchgrEcR*/*SchgrRXR.* After a boost injection (200 ng) on day 3, the locusts were sacrificed on day 6 to check the effect of RNAi-mediated knockdown of *SchgrEcR*/*SchgrRXR* on the transcript levels of *SchgrETHR* and *SchgrETHpre.* The knockdown efficiency of this experiment was previously published in Lenaerts *et al*,^29^. *SchgrETHR* and *SchgrETHpre* transcript levels were respectively 52% and 78% lower in the epidermis of *SchgrEcR/SchgrRXR* knockdown animals when compared to control animals (Fig. 7).

## Discussion

### Characteristics and expression patterns of *SchgrETHR* and *SchgrETHpre*

ETH precursor genes encode two peptides with ETH-like structure and biological activity. These peptides belong to the family of insect PRXamide peptides that consists of three subfamilies: 1) pyrokinins or pheromone biosynthesis activating neuropeptide (PBAN)-like peptides ending in –FXPRXa, 2) the CAP2b-like peptides ending in –FPRXa and 3) the ecdysis triggering hormones with the –PRXa sequence motif (as reviewed by Jurenka^27^). The latter are named pre-ETH and ETH in lepidopteran species and ETH1 and ETH2 in other species. The *Schistocerca* precursor sequence also encodes two ETH peptides. Identical peptides were identified earlier in a peptidomic survey of the *Locusta migratoria* neuroendocrine system using a combination of MALDI-TOF and ESI-Q-TOF mass spectrometry^30^ and later also confirmed in tracheal extracts of *Schistocerca americana* using immuno-isolated RP-HPLC-fractions and MALDI^22^.

ETH receptors are rhodopsin-like GPCRs and have been identified or predicted in different insect, tick and crustacean species^22^. A recent phylogeny of the PRXamide GPCRs in insects indicates that ETH receptors are basal with a considerable divergence time from the other PRXamide receptors^27^. Next to the conserved 7TM regions, *Schgr*ETHR possesses some typical features of the rhodopsin-like GPCRs, which are also conserved in vertebrate PRXamide receptors (indicated in Supplementary Figure S1). Also, two amino acids typically conserved in ETH receptors are shown, possibly indicating their importance in ligand interactions.

For the first time in a hemimetabolous insect, detailed tissue and temporal transcript profiles were determined for *SchgrETHR* and *SchgrETHpre*. The highest transcript levels for both genes were found in the trachea. It has been shown that the migratory locust, *L. migratoria,* possesses numerous small Inka cells scattered on the surface of broad and narrow trachea^22^. Inka cells are known to be the source of ETH in several insect species^18^. Therefore it is not surprising that even after removing trachea from the dissected tissues, Inka cells remain in these tissues and, as such, *SchgrETHpre* transcript levels can be measured. The fact that *SchgrETHR* is highly expressed in the trachea indicates a possible feedback of *Schgr*ETHs on their own transcription. Also in case of *SchgrETHR* it might be that the measured transcript levels in the other tissues are a consequence of remaining Inka cells. However, since the temporal profiles of *SchgrETHR* in the trachea and the epidermis are not similar, we believe that ETH and its receptor may not only play an important role in regulating the neuropeptidergic signalling cascade initiating ecdysis, as has been proven in several Holometabola^9^,^10^,^31^, but may also have a part in preparing the epidermis itself for ecdysis. Furthermore, the observed transcript levels of the receptor in the CA-CC complex, indicate that *Schgr*ETH might be involved in the release or even stimulation of production of neuropeptides from the CC, since these are obvious neurosecretory organs synthesizing and releasing different neuropeptides at different times during development^1^.

Similar to the situation in Holometabola^4^,^14^,^17^,^20^,^32^, the temporal distribution of *SchgrETHpre* in both fourth and fifth instars (Fig. 5) shows a clear correlation with the ecdysteroid titre; in *M. sexta* rising ecdysteroid titres result in enlargement of Inka cells and increased production of ETH^17^. Furthermore, it is known that the *eth* gene in *D. melanogaster* contains a direct repeat ecdysteroid response element^14^. Since the genome of *S. gregaria* has not been sequenced, we were not able to verify this. However, to further investigate if ETH acts indeed downstream of ecdysone, we studied the effect of the RNAi-mediated knockdown of the ecdysone receptor complex, *SchgrEcR/SchgrRXR*, on the transcript levels of *SchgrETH* (Fig. 7B). Transcript levels of *SchgrETH* were significantly reduced in *SchgrEcR/SchgrRXR* knockdown fifth instars, compared to control locusts. Based on research in Holometabola, the temporal distribution profile of *SchgrETHR* would also be expected to correlate with the ecdysteroid titre. In *M. sexta* and *A. aegypti* a peak in *ETHR* transcript levels is observed together with the peak in ecdysteroid titres^10^,^21^. A similar pattern is found in the epidermis of fifth instar locusts, with a peak on day 6 (Fig. 5). On the other hand, no clear correlation between ecdysteroid titres and ETHR transcript levels could be observed in the trachea and the brain. The relatively stable expression of *Schgr*ETHR in certain tissues might be a cost reduction measure since receptor protein synthesis would require more amino acids and cellular energy compared to the relatively shorter precursor for which expression is more dynamic (Fig. 5C,D). To further study if ETHR expression depends on ecdysteroid signalling, we performed an RNAi-mediated knockdown of *SchgrEcR*/*SchgrRXR* and checked the transcript levels of *SchgrETHR* in the epidermis of 6-day-old locusts. *SchgrETHR* transcript levels were significantly downregulated in the *SchgrEcR*/*SchgrRXR*-treated locusts when compared to control locusts. From our results it is clear that *SchgrETHpre*/*SchgrETHR* transcript levels are highly dependent on functional ecdysteroid signalling, since silencing the ecdysteroid heterodimer receptor transcripts results in significantly downregulated transcript levels of the ETH signalling system (Fig. 7A).

### Pharmacological profile *SchgrETHR*

Ligand binding at the extracellular surface of a GPCR will induce a change in conformation thereby activating a heterotrimeric G protein (G_αβγ_) which can subsequently trigger different cellular responses. Upon activation, the receptor can act as a guanine nucleotide exchange factor, inducing the exchange of GDP for GTP at the G protein. In an activated state, the α-subunit will associate with GTP, resulting in a dissociation of the G_αβγ_ complex. Classically these G proteins can be divided in four different families based on their G_α_ subunit: G_αs_, G_αi/o_, G_αq/11_ and G_α12/13_. Most functions of signal transduction by G proteins are realised by this subunit, with different G proteins interacting with different partners. As such, G_αs_ and G_αi/o_ were named for their potential to stimulate or inhibit adenylyl cyclase (AC), respectively, which will use ATP to synthesize cyclic AMP (cAMP) and further activate protein kinase A. G_αq/11_ will activate phospholipase C and couples the agonist-induced GPCR to the calcium pathway.

In determining the ligand of the *Schgr*ETHR, we cloned the receptor in CHO-WTA11 cells stably expressing apo-aequorin and the promiscuous Gα_16_ subunit. The cloned *Schgr*ETHR is activated by *Schgr*ETH1 and *Schgr*ETH2 in the nanomolar range. The first functional and pharmacological characterization of an ETH receptor was performed in *D. melanogaster* where the two splice variants were found to respond preferentially to ETH peptides, with *Drome*ETH1 being the most active ligand in *in vitro* studies^8^,^33^. The *Drosophila* splice variants showed some cross reactivity with related peptides displaying the –PRXamide signature. Pharmacological profiles of the recombinant *M. sexta* ETH receptor were also investigated in a similar CHO cell system, showing both *Manse*ETHR-A and -B to be highly specific for ETHs^10^. Also the *Aedes* ETH receptors were pharmacologically characterized using a CHO-K1 system without the promiscuous Gα_16_ subunit, thereby showing a clear mobilization of intracellular calcium stores upon receptor activation with different insect ETHs^21^.

We further investigated intracellular signalling of *Schgr*ETHR through the measurement of Ca^2+^ and cAMP responses in CHO-PAM28 cells expressing *Schgr*ETHR lacking the promiscuous Gα_16_ subunit or in HEK cells containing a cAMP response element (CRE)-dependent luciferase reporter construct. Our study indicated that *Schgr*ETHR displays dual coupling to both cyclic AMP and Ca^2+^ second messenger systems upon activation with *Schgr*ETH1 and *Schgr*ETH2. This new detailed information can be very beneficial in research focused on employing the ETH signalling pathway in pest management strategies. Although these data are indicative of the capacity of *Schgr*ETH-activated *Schgr*ETHR to couple to these second messengers, one cannot exclude at this point any possible discrepancies between cultured cell lines and intracellular processes occurring within different *S. gregaria* cell types *in vivo*.

### Physiological role of *SchgrETHR* and *SchgrETHpre*

The key role of ETH and its receptor in the moulting process has been proven in several Holometabola^2^,^4^,^7^,^8^,^10^,^14^,^16^,^21^,^32^. To our knowledge however, this role has not been confirmed in Hemimetabola. Using the major pest insect *S. gregaria*, our research proves that ETH is a crucial factor in the moulting process of Hemimetabola as well. RNAi-mediated knockdown of *SchgrETH pre* or *SchgrETHR* resulted in 100% and 88% lethality at the adult moult, respectively (Fig. 6). This not only points to a conserved role of ETH and its receptor, but also shows that this neuropeptide signalling system could be an excellent candidate target in the design of new pest control strategies.

In order to observe an effect of the RNAi-mediated downregulation of *SchgrETHR* on the adult moult, it was crucial to already initiate *SchgrETHR* dsRNA injections in fourth instars. When starting the treatment on the day of moulting to the fifth instar stage, no effect on the adult moult could be observed (results not shown), whereas a very strong and lethal phenotype could be observed when starting the dsRNA injections on day 1 of the fourth instar stage (Fig. 6A). An explanation for this might be that the knockdown efficiency is not strong enough when only starting injections at the beginning of the fifth instar stage. By looking at the knockdown efficiencies of *SchgrETHR* and *SchgrETH pre* in fourth and fifth instars (Suppl. Fig. S3), it is obvious that the efficiency of the RNAi-mediated knockdown is dependent on the gene of interest and that it is harder to effectively silence *SchgrETHR.* This is consistent with previous research in our lab, where we observed that some GPCR transcripts are more difficult to silence using RNAi, possibly due to their low mRNA levels in insect tissues and/or to the relatively slow turnover rate of GPCRs, ensuring the protein to remain present for a long time even after dsRNA injection (unpublished data). To cope with this problem, we tried injecting in an earlier stage which resulted in a significant improvement of the RNAi-mediated knockdown efficiency. Furthermore, we would like to mention that the timing of injection of dsRNA can also be critical. While trying to rescue the ETH knockdown phenotype in fourth instars using the synthetic peptide, we discovered that injecting dsRNA one day earlier can have big influence on the observed phenotype (Fig. 6A and C). It must be stressed that the *SchgrETHR* dsRNA construct was designed in the C-terminally situated region that is usually conserved in insect ETHR splice variants. However, we were unable to characterize a second isoform of *SchgrETHR* and therefore can neither confirm the existence of splice variants in *S. gregaria*, nor any possibly distinct functional roles as recently described for *Drosophila*^34^.

We were able to rescue 40% of the *SchgrETHpre* knockdown fourth instar locusts after several earlier unsuccessful rescue attempts (Fig. 6D). We believe the difficulties with rescuing the lethal phenotype lie in the variation of the exact timing of ecdysis behaviour, together with the lack of clear indicators determining pre-ecdysis and ecdysis in *S. gregaria*. Even though the exact behavioural sequence of moulting is known in *D. melanogaster*, Park and coworkers (2002) were also unsuccessful in rescuing all *eth* mutants. To our knowledge no other reports of successfully rescuing ETH knockdown or knockout insects exist.

## Conclusion

In conclusion, our data clearly indicate that ETH and its receptor are involved in the regulation of ecdysis in *Schistocerca gregaria*, thereby acting downstream of ecdysteroid signalling. The lethal phenotype observed upon silencing of *SchgrETHR* ensures it is an excellent candidate target for the development of locust management strategies. Furthermore, an *in vitro* study has shown the dual coupling characteristics of the receptor, affecting both Ca^2+^ and cAMP levels. Given the phylogenetic position of *S. gregaria*, these findings are a useful addition to our current knowledge and understanding of ecdysis, a crucial process for growth and development of insects, by far the largest class of animals on our planet.

## Materials and Methods

### Rearing of animals

The desert locusts, *S. gregaria*, were reared under crowded conditions (>200 locusts/cage) at constant temperature (32 ± 1 °C), constant day/night cycle (13:11 h photoperiod) and ambient relative humidity between 40% and 60%. The locusts were fed daily *ad libitum* with fresh cabbage leaves, supplemented with dry oat flakes. Following mating, females deposited their eggs in pots filled with a slightly moistened sand mixture (7 parts sand, 3 parts peat and 1 part water). Once a week these pots were collected and set apart in empty cages, where eggs were allowed to hatch into first instar larvae. In the described experiments, locusts were synchronized on the day of ecdysis into the fourth or fifth (final) instar stage. For the temporal expression profile of the genes of interest, locusts were dissected every day during the fourth instar stage and every other day during the fifth instar development. The setup (injection, dissection and observation) of the different RNAi experiments is described in each figure legend.

### Tissue collection

The locust tissues of interest were dissected under a binocular microscope and rinsed in locust Ringer solution (1 L: 8.766 g NaCl; 0.188 g CaCl_2_; 0.746 g KCl; 0.407 g MgCl_2_; 0.336 g NaHCO_3_; 30.807 g sucrose; 1.892 g trehalose). Tissues were immediately pooled in MagNA Lyser Green Beads Tubes (Roche) or RNase-free Screw Cap Microcentrifuge tubes and snap-frozen in liquid nitrogen to prevent RNA degradation. Tissues for the tissue and temporal expression profile of the genes of interest in fifth instars were collected in three pools consisting of five animals each. For the temporal expression profile in fourth instars the tissues were collected in six pools of three animals each. For the determination of the RNAi knockdown efficiency of *SchgrETHR* and *SchgrETHpre*, tissues were collected from 6 or 7 independent animals. To investigate the effect of the RNAi mediated knockdown of *SchgrEcR/SchgrRXR* on *SchgrETHR* and *SchgrETHpre*, tissues were collected in six independent pools of three animals each. Tissues were stored at −80 °C until further processing.

### RNA extraction and cDNA synthesis

Depending on the tissue, different RNA extraction methods were used. Brain and optic lobes, epidermis, fat body, Malpighian tubules, male gonads and female gonads were transferred to MagNA Lyser Green Beads Tubes (Roche) and homogenized using a MagNa Lyser instrument (1 min, 6500 rpm; Roche). Subsequently, total RNA was extracted from these tissue homogenates using the RNeasy Lipid Tissue Kit (Qiagen) according to the manufacturer’s protocol. A DNase treatment (RNase-Free DNase set, Qiagen) was performed to eliminate potential genomic DNA contamination. Because of the relatively small size of the prothoracic glands (PG), corpora allata (CA) and corpora cardiaca (CC), total RNA from these tissues was extracted using the RNAqueous-Micro Kit (Ambion) according to the manufacturer’s protocol. The manufacturer’s recommended DNase step was subsequently performed. Purity and concentration of the resulting RNA samples were checked using a Nanodrop spectrophotometer (Nanodrop ND-1000, Thermo Fisher Scientific, Inc.). For each RNA sample, cDNA was synthesized by reverse transcription of 500 ng of RNA with the PrimeScript™ RT reagent Kit (Perfect Real Time) (Takara, Invitrogen Life Technologies), using both random hexamer primers and oligo(dT) primers, according to the manufacturer’s protocol. The 10 μL reaction was diluted sixteen-fold with Milli-Q water (Millipore).

### Molecular cloning of *SchgrETHR* and *SchgrETHpre* and construction of the pc*Schgr*ETHR expression vector

Full length sequences for *Schgr*ETHR and *Schgr*ETHpre were found in the in-house *S. gregaria* transcriptome database. The complete ORF was PCR-amplified using fifth instar *S. gregaria* head cDNA and Q5^®^ High-Fidelity DNA Polymerase (New England Biolabs). A first PCR was performed with the primers listed in Table 1, after which the PCR product was purified using Sigma-Aldrich’s GenElute™ PCR Clean-Up kit, and subsequently used as a template in a nested reaction using the second primer set (Table 1). Amplicons were analysed on a 1.2% agarose gel, purified using the GenElute Gel Extraction Kit (Sigma-Aldrich), cloned into a pcDNA3.1/V5-His-TOPO TA expression vector (Invitrogen) and transformed into One Shot TOP10 chemically competent *E. coli* cells (Invitrogen). The sequence of the insert was confirmed by Sanger sequencing. Colonies harbouring the correct receptor insert were grown overnight in 100 ml Luria-Bertani medium and plasmid DNA was isolated using Qiagen’s EndoFree Plasmid Maxi Kit.

### Cell culture and transfection

Pharmacological studies were carried out in Chinese hamster ovary (CHO)-WTA11 stably expressing apo-aequorin, a zeocin resistance gene and the promiscuous Gα_16_ subunit coupling to the phospholipase C and Ca^2^^+^ signalling cascade (Euroscreen, Belgium). CHO-PAM28 cells (stably expressing apo-aequorin and a puromycin resistance gene) and human embryonic kidney (HEK) 293 T cells were used to assess effects on the Ca^2+^ and/or cAMP second messenger systems, respectively. CHO cell lines were provided by Prof. Marc Parmentier (University of Brussels, Belgium) and Dr. Michel Detheux (Euroscreen S.A., Belgium).

All cells were cultured *in vitro* as monolayer at 37 °C with a constant supply of 5% CO_2_ in Dulbecco’s Modified Eagles Medium nutrient mixture F12-Ham (DMEM/F12, Invitrogen) supplemented with 1% penicillin/streptomycin (10000 units/ml penicillin and 10 mg/ml streptomycin in 0.9% NaCl, Invitrogen) in order to prevent bacterial contamination and with 10% of fetal bovine serum (Sigma-Aldrich). CHO-WTA11 and CHO-PAM28 cell medium was supplemented with 250 μl/ml of zeocin (Invitrogen) and 5 μl/ml puromycin respectively. HEK293T cell medium was supplemented with 2% Ultroser G medium (Pall Life Sciences). Cells were subcultivated twice a week.

Transfections with the plasmid DNA of *SchgrETHR* were carried out in T75 flasks at 60–80% confluency. Transfection medium consisted of 2.5 ml of Opti-MEM (Invitrogen), 10 μg plasmid DNA, 12.5 μl Plus^TM^ Reagent (Invitrogen) and 30 μl of Lipofectamine^TM^ LTX (Invitrogen). After incubation at room temperature, the mix was drippled onto the cells and supplemented with 3 ml of fresh cell culture medium (as described above). HEK293T cells were co-transfected with 5 μg of *SchgrETHR* plasmid DNA and 3 μg of a CRE-luciferase reporter construct (containing the ORF of luciferase downstream of a multimerised cAMP-response-element^35^.

### Calcium reporter assay in CHO cells

CHO cells were detached from the flask using phosphate buffered saline (PBS) supplemented with 0.2% EDTA (pH 8.0), collected in DMEM/F-12 (without phenol red, with L-Gln and 10 mM HEPES; Gibco) and pelleted for 4 min at 800 rpm at room temperature. The amount of live cells per ml was measured using the TC20™ Automated Cell Counter (Bio-Rad). The pellet was resuspended in BSA-medium (DMEM/F-12 without phenol red, containing L-glutamine and 10 mM HEPES and supplemented with 0.1% bovine serum albumin) to a final concentration of 5 × 10^6^ cells/ml. Coelenterazine H (Invitrogen) was added to a final concentration of 5 μM, and cells were gently shaken at room temperature in the dark for 3 h. *Schgr*ETH1 and *Schgr*ETH2 peptides were dissolved in BSA-medium and a dilution series was prepared. 50 μl of the final ligand concentration was pipetted into a 96-well plate. Wells containing 50 μl of BSA-medium without ligand were used as a negative control. Activity of the recombinant receptor was measured as the light emitted for 30 s after addition of 50 μl of the cell suspension using a Mithras LB940 (Berthold Technologies). Cells were lysed with Triton X-100 (0.2% in BSA-medium) and light emission was recorded for another 8 s. Light emission from each well was normalized to the total response (ligand + Triton X-100) using the Mikrowin2000 software (Mikrotek). Further analysis was performed using GraphPad Prism 6 (GraphPad Software Inc.).

### Cyclic AMP (cAMP) reporter assay in HEK293T cells

Cotransfected HEK293T cells were detached, counted and pelleted as described above. Cells were resuspended at a concentration of 10^6^ cells/ml in DMEM/F-12 supplemented with 200 μM IBMX for studying stimulatory effects or supplemented with 200 μM IBMX and 20 μM NKH-477 (H_2_O: soluble analogue of forskolin; Sigma-Aldrich) to study inhibitory effects. DMEM/F-12 supplemented with 200 μM IBMX was used as negative control, DMEM/F-12 containing 200 μM IBMX and 20 μM NKH-477 was used as positive control. Dilution series were made for the *Schgr*ETH peptides. 50 μl of peptide was added to the wells of the 96-well plate already containing 50 μl of cells suspension. The plate was subsequently incubated for 3–4 h at 37 °C and 5% CO_2_. 100 μl of SteadyLite Plus (Perkin Elmer) was added to the wells of the plate, after which the plate was gently shaken at room temperature for 15 min in the dark. Luciferase activity in the shape of light emission was measured for 5 s/well using a Mithras LB940 (Berthold Technologies). Data were analysed as described above.

### Quantitative real-time PCR (qRT-PCR)

Primers used in the qRT-PCR profiling are given in Table 2. These primers were validated by designing relative standard curves for gene transcripts with serial dilutions (5x) of fifth instar brain cDNA. All qRT-PCR reactions were performed in duplicate in 96-well plates on a StepOne System (ABI Prism, Applied Biosystems). Each reaction contained 5 μl Fast SYBR^®^ Green Master Mix (Applied Biosystems), 0.5 μL forward and reverse primer (10 μM) and 4 μL previously diluted cDNA. Following thermal cycling profile was used: 95 °C for 10 min, followed by 40 cycles of 95 °C for 15 s and 60 °C for 60 s. Finally, a dissociation protocol was performed, allowing melt curve analysis to check for primer dimers. Only a single melting peak was found for all transcripts. Additionally, amplification products were analysed using horizontal agarose gel electrophoresis and visualized using UV. Only a single band could be seen which was further cloned and sequenced (TOPO^®^ TA cloning kit for sequencing, Invitrogen) to confirm target specificity. Suitable reference genes were selected from a pool of candidate reference genes by means of the geNorm software^36^,^37^. Randomly selected cDNA samples were used to determine the optimal reference genes. For the temporal distribution profiles in the heads of fourth instars and the epidermis of fifth instars, as well as for the tissue distribution profiles in fifth instars, *RP49* and *EF1α* were selected by the geNorm software as having the most stable expression. For the temporal distribution profiles in the brain of fifth instar *GAPDH* and *CG13220* were selected by the geNorm software as having the most stable expression, while *α-tubulin1A, β-actin* and *EF1α* were selected for the temporal distribution profiles in the trachea. All qRT-PCR results were normalized to the transcript levels of the selected reference genes and calculated relative to the transcript level in a calibrator sample according to the comparative Ct method. qRT-PCR was used to determine the tissue and temporal distributions of genes of interest in the last instar stage, therefore cDNA samples were used from fifth instar male locusts, except the female gonads. GraphPad Prism 6 (GraphPad Software Inc.) was used to test the statistical significance of the observed differences for the RNAi experiments. Normalized relative quantities were log-transformed.

### Ecdysteroid measurements using an enzyme immunoassay (EIA)

Ecdysteroid titres in *S. gregaria* hemolymph were measured using an enzyme immunoassay, modified from Porcheron *et al*.^38^ and discussed by Pascual *et al*.^39^ and Lafont *et al*.^40^. In this protocol a peroxidase conjugate of 20E was used as tracer together with rabbit L2 polyclonal antibodies. This L2 antiserum has a strong affinity for E, 3-deoxyecdysone and 2-deoxyecdysone and a 6- to 8-fold lower affinity for 20E. Both serum and tracer were very kindly given by Prof. J.P. Delbecque (Université de Bordeaux, France). Hemolymph samples were collected from *S. gregari*a fourth or fifth instar locusts by piercing the insect’s cuticle behind its hind leg and holding a capillary to the wound. For the temporal ecdysteroid titre profile hemolymph was pooled in five groups of three locusts each. 10 μL of hemolymph was collected from each animal, which was immediately transferred to 270 μL of cold methanol (100%) and stored at −20 °C until further processing, as described by Marchal *et al*.^41^. The standard curve used in all measurements was obtained with 20E, and therefore results are expressed as 20E equivalents.

### RNA interference experiments

#### Production of dsRNA

dsRNA constructs for *SchgrETHR, SchgrETHpre, SchgrEcR* and *SchgrRXR* were produced using the MEGAscript^®^ RNAi Kit (Ambion) according to the manufacturer’s protocol. This procedure is based on the high-yield transcription reaction of a user-provided linear transcript with a T7 promoter sequence. Forward and reverse primers flanked by the T7 promoter sequence were (Table 3) used in a PCR reaction with REDTaq^®^ DNA polymerase (Sigma-Aldrich Co.) to amplify a fragment of the target gene (200–600 bp). Both dsRNA constructs for *SchgrEcR* as well as *SchgrRXR* were designed against the common region of their isoforms^29^. PCR products were analysed using horizontal agarose gel electrophoresis and visualized using UV. This resulted in bands of 404 bp, 253 bp, 304 bp and 307 bp for respectively *SchgrETHR, SchgrETHpre, SchgrEcR* and *SchgrRXR.* These bands were further cloned and sequenced (TOPO^®^ TA cloning kit for sequencing, Invitrogen) to confirm the amplicon sequence. The purity and concentration of the produced dsRNA was determined by means of spectrophotometry (Nanodrop ND-1000). To confirm dsRNA integrity, a small amount of the reaction product was checked on an agarose gel.

#### RNAi experiment

To investigate the knockdown efficiency of *SchgrETHR* and *SchgrEHTpre*, one-day-old fourth instars were injected with dsRNA against *SchgrETHR* or *SchgrETHpre* (400 ng in 5 μL locust Ringer solution). A boost injection was given on day 4 of the fourth instar stage, as well as on day 1, 3 and 5 of the fifth instar stage. Control locusts were injected with a *GFP* dsRNA construct (400 ng in 5 μL locust Ringer solution). Part of these locusts were dissected on day 4 of the fourth instar stage, the other part on day 6 of the fifth instar stage. To investigate the effect of an RNAi-mediated knockdown of *SchgrETHR* or *SchgrETHpre* on moulting different injections schemes were applied, as described in Fig. 6. To investigate the effect of an RNAi-mediated knockdown of the ecdysone receptor complex, *SchgrEcR/SchgrRXR*, on the transcript levels of *SchgrETHR* and *SchgrETHpre*, we used samples from an earlier published experiment^29^. There newly moulted fifth instars were injected with a mix of *SchgrEcR* and *SchgrRXR* dsRNA construct (200 ng each in 8 μL locust Ringer solution). A second injection was given three days after moulting. Control locusts were injected with a *GFP* dsRNA construct (200 ng in 8 μL locust Ringer solution). Locusts were then dissected on day 6 to check the effect of the knockdown on the transcript levels of *SchgrETHR* and *SchgrETHpre*.

## Additional Information

**How to cite this article**: Lenaerts, C. *et al*. The ecdysis triggering hormone system is essential for successful moulting of a major hemimetabolous pest insect, *Schistocerca gregaria. Sci. Rep.* **7**, 46502; doi: 10.1038/srep46502 (2017).

**Publisher's note:** Springer Nature remains neutral with regard to jurisdictional claims in published maps and institutional affiliations.

## Supplementary Material

Supplementary Figures

## Figures and Tables

**Table 1:** Oligonucleotide sequences for primers used in cloning and sequence analysis of *SchgrEcRA, SchgrEcRB1* and *SchgrRXR*.

	Forward primer	Reverse primer
*SchgrETHR*	5′-CGGCTCCTTTGATGAATCTCG-3′	5′-GTCACCAGTATACGAGGATG-3′
*SchgrETHR_nested*	5′-CACCATGAATCTCGCGCTGTC-3′	5′-TCACAGGTAGGAGACGTCC-3′
*SchgrETHpre*	5′-ACGCAGGAAGGAACGAGTC-3′	5′-TCTTTCCGGAGTTCAACGAC-3′
*SchgrETHpre_nested*	5′-ATGCTGCTCTTCAAGGAGACTT-3′	5′-CTATCGTGAGTTATCAACTTCCCG-3′

Abbreviations: *ETHR* = *ecdysis triggering hormone receptor, ETHpre* = *ecdysis triggering hormone precursor. A partial Kozak sequence (CACC*) *was added to the 5*′ *end of the SchgrETHR_nested forward primer to allow initiation of translation in mammalian cells.*

**Table 2:** Oligonucleotide sequences for primers used in qRT-PCR.

Reference genes	Forward primer	Reverse primer
*β-actin*	5′-AATTACCATTGGTAACGAGCGATT-3′	5′-TGCTTCCATACCCAGGAATGA-3′
*EF1α*	5′-GATGCTCCAGGCCACAGAGA-3′	5′-TGCACAGTCGGCCTGTGAT-3′
*RP49*	5′-CGCTACAAGAAGCTTAAGAGGTCAT-3′	5′-CCTACGGCGCACTCTGTTG-3′
*CG13220*	5′-TGTTCAGTTTTGGCTCTGTTCTGA-3′	5′-ACTGTTCTCCGGCAGAATGC-3′
*α-tubulin1A*	*5′-TGACAATGAGGCCATCTATG-*3′	*5′-TGCTTCCATACCCAGGAATGA-*3′
*GAPDH*	5′-GTCTGATGACAACAGTGCAT-3′	5′-GTCCATCACGCCACAACTTTC-3′
**Target genes**	**Forward primer**	**Reverse primer**
*SchgrETHR*	5′-CCAACCCGCGCATCAT-3′	5′-CCTGCTTGCGGTACCTGTAGT-3′
*SchgrETHpre*	5′-GACGAGGGAGCCAATTTGT-3′	5′-GATTTGGCGCTCTTGAGG-3′

Abbreviations: EF1*α* = *elongation factor 1 alpha, RP49* = *ribosomal protein 49, GAPDH* = *glyceraldehyde-3-phosphate dehydrogenase, ETHR* = *ecdysis triggering hormone receptor, ETHpre* = *ecdysis triggering hormone precursor.*

**Table 3:** Oligonucleotide sequences for primers used in dsRNA construct design.

Target genes	Forward primer	Reverse primer
*SchgrETHR*	5′-TAATACGACTCACTATAGGGAGA CGTCATCATCATGAGCATC-3′	5′-TAATACGACTCACTATAGGGAGA GGCGAGCAGACAGATGAGC-3′
*SchgrETHpre*	5′-TAATACGACTCACTATAGGGAGA TGGTCCTGGTGGCGGCA-3′	5′-TAATACGACTCACTATAGGGAGA TTTGTGCGGCGGCCAATC-3′
*SchgrEcR*	5′-GAAATTAATACGACTCACTATAGGGCC ACGTGAGGTTTCGGCACATC-3′	5′-GAAATTAATACGACTCACTATAGGGCC GTTTCCCCCATACCAGCCAG-3′
*SchgrRXR*	5′-GAAATTAATACGACTCACTATAGGGCC GCTCAATGGGTCCACAGTCA-3′	5′-GAAATTAATACGACTCACTATAGGGCC ACACCATAATGCTTCCCGCT-3′
*GFP*	5′-TAATACGACTCACTATAGGGAGA AAGGTGATGCTACATACGGAA-3′	5′-TAATACGACTCACTATAGGGAGA ATCCCAGCAGCAGTTACAAAC-3′

Underlined sequences are the T7 promoter sequences.

Abbreviations: *ETHR* = *ecdysis triggering hormone receptor, ETHpre* = *ecdysis triggering hormone precursor, EcR* = *ecdysone receptor, RXR* = *retinoid-X-receptor, GFP* = *green fluorescent protein.*
