# Supplementary material for: The ecdysis triggering hormone system is essential for successful moulting of a major hemimetabolous pest insect, Schistocerca gregaria
Source: Sci Rep. 2017 Apr 18;7:46502. doi: 10.1038/srep46502 (PMC5394484; doi:10.1038/srep46502)
Supplement: Supplementary Figures [file srep46502-s1.pdf]

The ecdysis triggering hormone system is essential for successful moulting of a major hemimetabolous pest insect, *Schistocerca gregaria*.

Cynthia Lenaerts[1], Dorien Cools[1], Rik Verdonck[1], Lina Verbakel[1], Jozef Vanden Broeck[1], Elisabeth Marchal[1*]

Supplementary figures

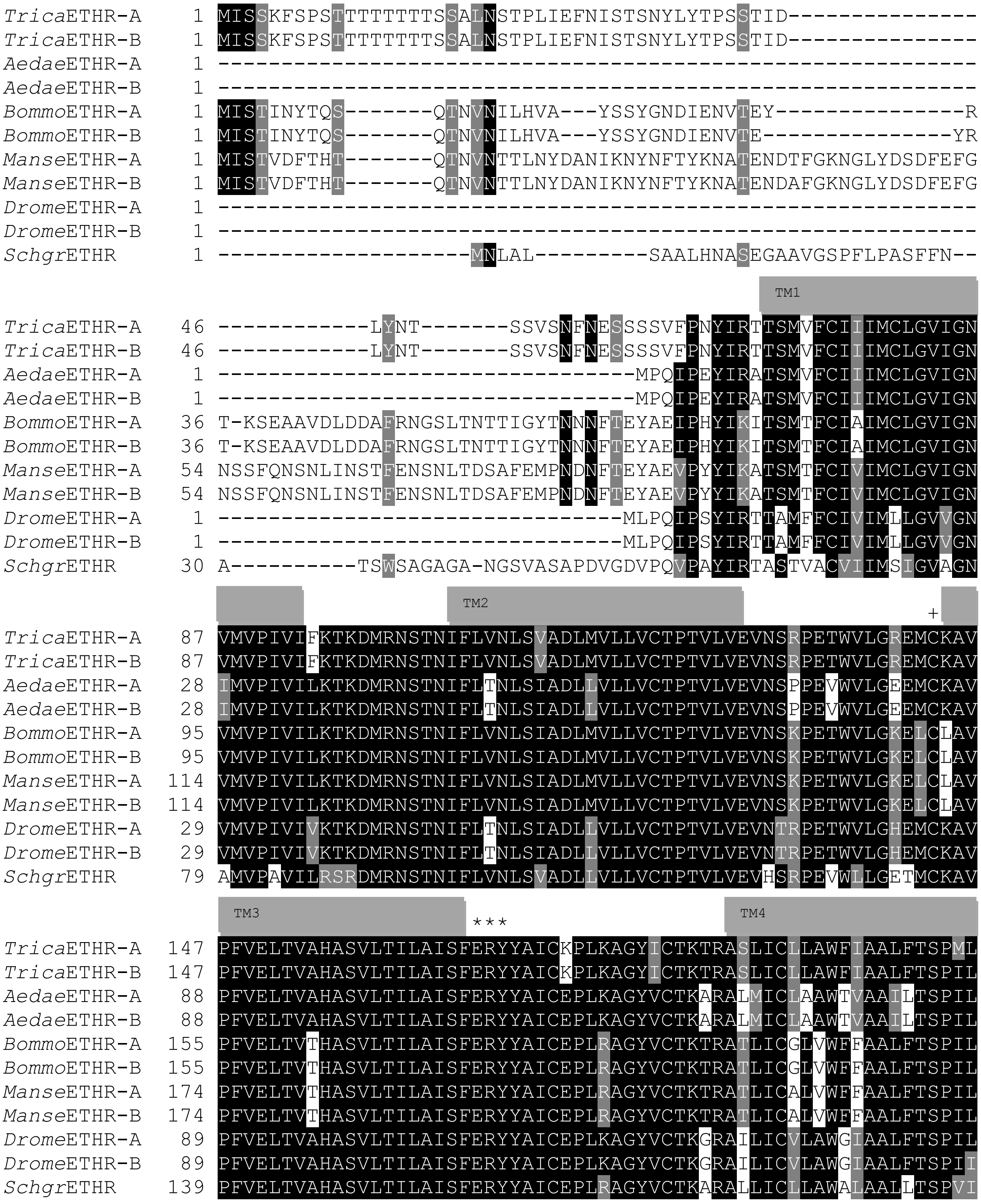

```
                                                     TM5
                                +
TricaETHR-A  207 VIAEYKQLDYFDGSKVPACHTLANTFWSALYFLTIIFLFFIIPLIILLILYCIIAKNLMS
TricaETHR-B  207 AITQYGPEEYVDGSIVFVCFSLVEDTLPCVFFLGSILIFFIFPLAILIIVYILIAKTLMH
AedaeETHR-A  148 LFAEYSVEEYPDGSRAAVCLTKASNVWTVTFFLMTISLFFLLPLVILVVLYAIIAKNLIA
AedaeETHR-B  148 MISSYEYVEYVNGKMVASCLTPVENFWHATFFVASIVVFFIVPLLILVVLYSVIAKNLMD
BommoETHR-A  215 AIADHKA-TSVNGTVVNQCLTQAGTVWEITFFVTIIILLYLLPLIILIVLYSIIAKNLIT
BommoETHR-B  215 AVATFTYEQDEDGTEVPVCLTQADTFWSALFFILTIAIFFIVPLGVLLVLYSVIAKNLME
ManseETHR-A  234 AIAEHHTIVHHDGTTTSQCLTQAVTFWQISFFVMIIILLYILPLIILIVLYSVIAKNLIT
ManseETHR-B  234 GVAKFTYEKYSDGTEVPVCLTQADTFWSALFFILTIAVFFIVPLGVLLVLYSVIAKNLME
DromeETHR-A  149 WVAEYKLAEYIDGSSVAVCLTQAISDWTLAFFLMTISVFFVVPFVTLVVLYGIIARNLVS
DromeETHR-B  149 AISTYSVEPYGDGTDAPVCTTAADGFWSIFYFVGCITVFFFLPFGILVLLYAAIAYKLLR
SchgrETHR    199 LVAEYGTEEYFDGSMVPVCFSMVDSFWPAFFFLATIAVFFLVPLIILVVLYGVIARHLMA

                                                                 TM6
TricaETHR-A  267 NAATLVLNKH-I-------------------------DNISLRARKQVVLMLGTVVLSFF
TricaETHR-B  267 HPVAMTGTKT-V-----------------------MPSQSAIKYRKQVILMLGTVVLAFF
AedaeETHR-A  208 SNNSRIKIRLSKPE-------------------------LSYKARKQVVLMLGAVVLAFF
AedaeETHR-B  208 NPGIIMSNASG-------------------------NRNNVIKYRKQVIFMLGAVVVSFF
BommoETHR-A  274 AASKVVMNK--------------------------TVDPYNARARKQVILMLGTVVLCFF
BommoETHR-B  275 NPVIIAQSSKNT-----------------------SGTGNVIRYRKQVILMLGTVVLSFF
ManseETHR-A  294 AASKVVMNK--------------------------TVDPYNARARKQVILMLGTVVACFF
ManseETHR-B  294 NPAIIAQNNK-N-----------------------SGSGNVVRYRKQVILMLGTVVLSFF
DromeETHR-A  209 NRAAMLRARPTKPE-------------------------LSLKARKQVVLMLGAVVLSFF
DromeETHR-B  209 PNNAFHRPTSPQPQQPSGGATSGSSQVPSTKGNSHQQSNGMRKHRKQVIFMLVAVVSSFF
SchgrETHR    259 NPRIMSV-----------------------------HQQLHYRYRKQVVLMLGTVVAAFF

                  O                                 TM7
TricaETHR-A  301 LCLIPFRVFILWIILVPEEQVYHLEIEKYYNILYFCRIMVYLNSAINPILYNLMSSKFRT
TricaETHR-B  303 ICLLPFRALTFWIIVAPAGSNFEIGFENYYNILYFSRIMFHINSAVNPILYNIISSKFRS
AedaeETHR-A  243 TCLLPFRMLTLWIIMVSEETFQKLAVEKYYNLLYFSRIMLYLNSAVNPILYNLMSSKFRK
AedaeETHR-B  243 FCLLPFRALTLWIIIVPSENIISIGIERFYILLYFCRIMLYMNSAINPILYNLMSSKFRN
BommoETHR-A  308 LCLMPYRALTLWIIITPS-GFDGISSEKWYNILYFSRVMLYINSAINPILYNLMSSKFRI
BommoETHR-B  312 ICLLPFKALTLWIIVFPPETIMSLGIDGYYILLYFCRVMLYLNSAINPILYNLMSSKFRD
ManseETHR-A  328 LCLMPYRALTLWIIITPT-GFDDVSSEKWYNILYFSRVMLYINSAINPILYNLMSSKFRI
ManseETHR-B  330 ICLLPFKALTLWIIIFPPETIMSLGIDGYYILLYFCRVMLYLNSAINPILYNLMSSKFRE
DromeETHR-A  244 VCLLPFRVLTLWIILSTDQTLHDLGLVRYYSLLYFCRIMLYLNSAMNPILYNLMSTKFRR
DromeETHR-B  269 VCLLPFRAFTLWVILASAEDVEGLGIAGYYNLLYFSRFMLYLNSAMNPILYNLMSSKFRS
SchgrETHR    290 VCLLPFRAFTLWIIAAPPETVVSIGYENHYGLLCLCRVMHYLNSAVNPILYNLMSSKFRD

TricaETHR-A  361 GFIICSESN-RKF-----YFKRARNGT--------FSTT------ANSC-----------
TricaETHR-B  363 GFFKLCGMKIVKK-----RRKDKREIT--------RKSTSSSTHTSSQQ-----------
AedaeETHR-A  303 GFLRLCRCRRLWGHP--LR----RRGGKVRG----RSATFTTT-----------------
AedaeETHR-B  303 GFLQLLGCGKIIRSD--SISSGARKGT-----FHTASTNLSSSHNS---SYQRRFTREGS
BommoETHR-A  367 GFCKVCICYKK---ENDLNRRTQRTIT--------NGSTTSSSLTR--------------
BommoETHR-B  372 GFVKLLKINKLMRCSRNLRETMQRRDT--------FNTTTSTGFSSSQN-----------
ManseETHR-A  387 GFCKVCICYKK---EEELNRRAQRTIT--------NGSTTSSSLTR--------------
ManseETHR-B  390 GFVKLLKINKLMRYSRNLRETMQRRDT--------FNTTTSTGFSSSQN-----------
DromeETHR-A  304 GFKRLCQDAGRLLLE--LVTLGRRKEDSSRG----RRGTLSLGMGTNTNTNTNSSNATGA
DromeETHR-B  329 GFWRLLLT-----------CLGQRPHHHHRHHYHQRQHPTAGGSGRNASTRQEQDAEEGA
SchgrETHR    350 GFSRLCCRCGCAGGP--LGRRGTGLSSSATA------TTVRSSAGDSLW------RRPGR

TricaETHR-A  390 ----RSSTFRNNR--------DEYNVCFRPRNNSILIKNFNDS---------------PE
TricaETHR-B  399 ----TSDSFLSKR--------NSEKKCGSLRE----IKEVSDCD-------------SSE
AedaeETHR-A  336 ----TTSSYLASSSIRKSS-----------------EKYTLSLDDLRFQQLPVCSNGL-P
AedaeETHR-B  353 CISGSIRAVSDN-SRRR----LLHQRHS---------------------------STISI
BommoETHR-A  402 ----TTNSLKKFFGHRTSVDRSEAETNSKDEERSLFDRIFPNRAFLRQQSAPVCS--NLN
BommoETHR-B  413 ----TSDSFWRRYSNRVSSQKNILNNSKKIKEE----KV---------NPIKIGEIINVE
ManseETHR-A  422 ----TTNSLKKLFSHRGSVERSEIESCNREEEKGLFERIFPNRSFIRQQSAPVCS--SNN
ManseETHR-B  431 ----TSDSFWRRYSNRTSSQKIILNKERLQDTE----RK---------QSLKIGEIVNVE
DromeETHR-A  358 ----TSSSILSRSSNRRCS-----------------EDIS--RTRLKIEMQMPCGSDLEA
DromeETHR-B  378 ALAGTTSARHPRRTLRREATFLINSISTSSG-----TDRTTSSSAWRSNSLS-----ISG
SchgrETHR    396 A---SSSSSRSRRAERR----LLHHIA------------------------RECSVSVHN
```

```
TricaETHR-A  423 --SNRSNE----------------------------------------------------
TricaETHR-B  430 --SRKEVY----------------------------------------------------
AedaeETHR-A  374 -MASTESGTRGKDASSIVVVVSDDD--EEPFQLSRFYRMNLLRQYSTPLLPCYEDRGRPP
AedaeETHR-B  381 RSSHDEHSGTGGGGSGVPIQTGNGTIETTPLKADKIEELD-----------------ETT
BommoETHR-A  456 --PNRINRMRSEGC----------------MDINRPDNI-----HS--NLNPKVIRSEID
BommoETHR-B  456 --NTRRNSMKFIAA----------------LNED-AQIDNEVEIAD--NENNKQIQ----
ManseETHR-A  476 --PNKINRMRSEGC----------------MEIDKIEDVGRVHSNS--NFNNKNIRSSVD
ManseETHR-B  474 --NTRRNSMKVIAA----------------LNES-IEAVDEA-SEY--TEHNKQIH----
DromeETHR-A  395 -MAMLQHSTLGKGIA---RRVSDSR--LMPL------------------RNHQPRRHKPQ
DromeETHR-B  428 -LSERERGILGAAIIGTTA--------------------------ATVTTACLQERRASK
SchgrETHR    425 GVCAVKA-GRSKDVSYL*------------------------------------------

TricaETHR-A  429 IDGPEK*-----------------------------------------------------
TricaETHR-B  436 VRVPLGQRFSNGEIFV*-------------------------------------------
AedaeETHR-A  431 LPSTEPSGINS--------------------------NKSVESAV--KRQAFNVTFKESE
AedaeETHR-B  424 NSLPNGYGFNR--------------------------FKHVASAA--GGNSCNVSFKTTN
BommoETHR-A  491 ADLPRANSLRRNVLINTAKAKSVDSERNVMNYSKKTKVDSVVAFQK----SKSVDYEFPE
BommoETHR-B  491 ------------ILNLDVKTNSVYSITLDVSKEGKNRFVCVPAQDRDNKNIFIYDYNTKE
ManseETHR-A  516 LDMPRANSLRRSVLINSAKAKSVDSDKKFVSYQ-KIKVDCVVAFQK----SKSVDYEFPE
ManseETHR-B  508 ------------ILNLDKKTNSVYSITLDVNNEGGNRFMCIPQDAEYKNNIYICNYNAKE
DromeETHR-A  431 IS-FDEESLEE--------------------------NKRSEAKI--PTK-------CRE
DromeETHR-B  461 I*----------------------------------------------------------
SchgrETHR        ------------------------------------------------------------

TricaETHR-A      ------------------------------------------------------------
TricaETHR-B      ------------------------------------------------------------
AedaeETHR-A  463 RRKNNNNDSAGSNGVILVEPGSAWGNLFNPW-------------------KLKFIRQQTT
AedaeETHR-B  456 SCN--GKTSHRMNGTVSVEPKAVHQA-DNKYLIVRAAVVVMREVSEENGKPLPLSSSTKS
BommoETHR-A  547 SFV*--------------------------------------------------------
BommoETHR-B  539 SFV*--------------------------------------------------------
ManseETHR-A  571 SFV*--------------------------------------------------------
ManseETHR-B  556 SFV*--------------------------------------------------------
DromeETHR-A  455 -------KLPGIAREIVNLT----ENTL*-------------------------------
DromeETHR-B      ------------------------------------------------------------
SchgrETHR        ------------------------------------------------------------

TricaETHR-A      ------------------------------------------------------------
TricaETHR-B      ------------------------------------------------------------
AedaeETHR-A  504 PPQLQEQRPNSHVASAKAAVIIGVERRLPMSPRTKKQM-SLDESLLGARGNHCTVEGD*-
AedaeETHR-B  513 PPAENGST------EAEGT-LI-------QSARGSEQIVSIITAKERANG-NCVNRTQRA
BommoETHR-A      ------------------------------------------------------------
BommoETHR-B      ------------------------------------------------------------
ManseETHR-A      ------------------------------------------------------------
ManseETHR-B      ------------------------------------------------------------
DromeETHR-A      ------------------------------------------------------------
DromeETHR-B      ------------------------------------------------------------
SchgrETHR        ------------------------------------------------------------

TricaETHR-A      ------------------------------
TricaETHR-B      ------------------------------
AedaeETHR-A  561 ---------SGAGEV*--------------
AedaeETHR-B  558 DTKNTNEDAVAFDEINKIRQFYGSMECDI*
BommoETHR-A      ------------------------------
BommoETHR-B      ------------------------------
ManseETHR-A      ------------------------------
ManseETHR-B      ------------------------------
DromeETHR-A      ------------------------------
DromeETHR-B      ------------------------------
SchgrETHR        ------------------------------
```

Supplementary figure S1. Multiple sequence alignment of the cloned *Schgr*ETHR with other functionally characterized holometabolous insect ETH receptors (Clustal Omega, http://www.ebi.ac.uk/Tools/msa/clustalo). The seven transmembrane regions (as predicted for *Schgr*ETHR) are indicated above the sequences in grey shading. A disulphide bridge highly conserved in rhodopsin-like GPCRs, indicated with "+" is formed between a cysteine (C) at the beginning of transmembrane 3 and in extracellular loop 2. The histidine (H) typically conserved in transmembrane region 3 of all ETH receptors is indicated with "◊". The typical rhodopsin-like conserved ERY motif immediately following the third transmembrane helix is indicated with asterisks (***). The leucine (L) replacing tryptophan (W), which is a typical feature of ETH receptors, in the highly conserved FXXCWXPFHXXR motif in transmembrane 6 of rhodopsin-related GPCRs is indicated with "**O**".

>*Schgr*ETH

*MLLFKETFASLAVLVVLVAAAAA*EPDEGANLFLKASRSVPHV**GRR**SDFFLKTAKSVPRI**GRR**SDLFLKSAKSVPRI**GRR**TNLAAI EAQDGSEWLWPGGADAMPAPV**RR**QAYYVRKDGQPVMWSDVARDVEENPDLWPWNDFDAGNTREVDNSR*

Supplementary figure S2. Translation of the cloned *Schgr*ETH precursor sequence. The signal peptide as predicted by SignalP 4.1, is indicated in italics and underlined. *Schgr*ETH1 and *Schgr*ETH2 are boxed. Cleavage sites are indicated in bold. * represents a stop codon.

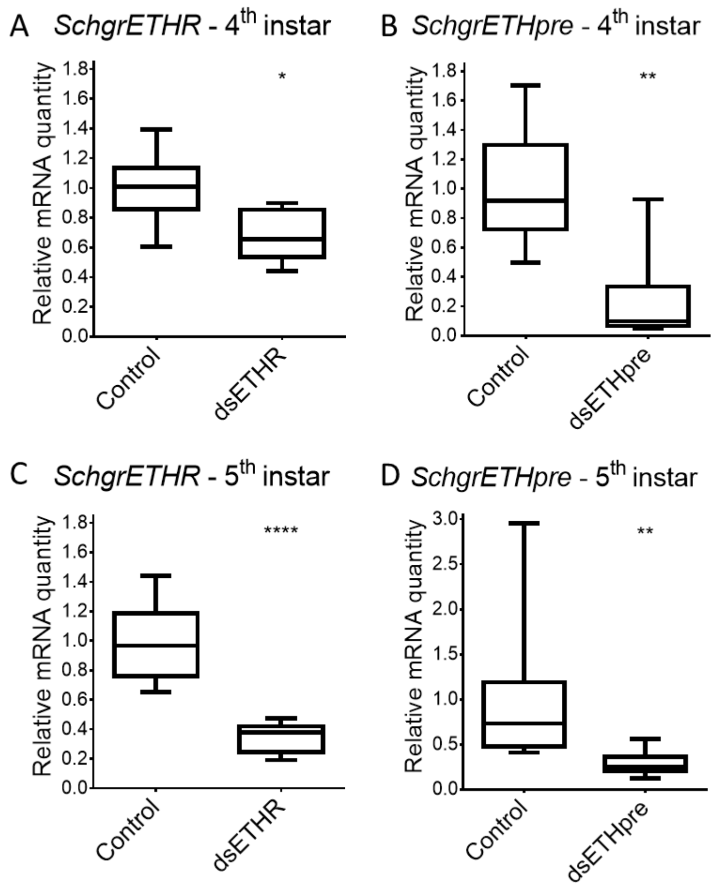

Supplementary figure S3. Efficiency of RNAi-mediated knockdown of *SchgrETHR* and *SchgrETHpre* in (A-B) 4-day-old fourth instar and (C-D) 6-day-old fifth instar male *S. gregaria.* Relative *SchgrETHR* and *SchgrETHpre* transcript levels were measured in the heads dissected from control locusts and locusts injected with *SchgrETHR* or *SchgrETHpre* dsRNA, using qRT-PCR. Locusts were injected on day 1 and 4 of the fourth instar stage, as well as on day 1, 3 and 5 of the fifth instar stage with 400 ng of dsRNA against *GFP* (control), *SchgrETHR* or *SchgrETHpre*. The data represent box plots (min to max) of six or seven individual locusts, run in duplicate and normalized to *RP49* and *EF1α* transcript levels for fourth instars and *β-actin* and *EF1α* transcript levels for fifth instars. Significant differences ($p < 0.5$, $p < 0.01$ and $p < 0.0001$) are indicated by (an) asterisk(s) (*, ** and **** respectively) (one-sided Welch's *t*-test on log-transformed data).
